# Supplementary material for: MAPK ERK5 is a novel regulator of MHC-I in cancer cells
Source: Cell Commun Signal. 2026 Mar 9;24:227. doi: 10.1186/s12964-026-02780-9 (PMC13085394; doi:10.1186/s12964-026-02780-9)
Supplement: Supplementary file 2 — Supplementary Material 2. [file 12964_2026_2780_MOESM2_ESM.pdf]

Supplementary Table 1. Primary and secondary antibodies used in immunoblot analyses.

**Antibody**	**Source**	**Reference**	**Dilution**	**Host**
MHC-I	Cell Signaling	35923	1:2000	Rabbit
p21	Millipore	05-655	1:1000	Mouse
p-ERK5	Cell Signaling	3371	1:1000	Mouse
ERK5	Cell Signaling	3372	1:1000	Rabbit
LC3I/II	Cell Signaling	12741	1:2000	Rabbit
p-ERK1/2	Cell Signaling	4376	1:1000	Rabbit
ERK1/2	Cell Signaling	4695	1:8000	Rabbit
p-Akt (S473)	Cell Signaling	9271	1:1000	Rabbit
Akt	Cell Signaling	9272	1:5000	Rabbit
c-Myc	Cell Signaling	9402	1:5000	Rabbit
Hsp90 beta	Invitrogen	PA3-012	1:16000	Rabbit
GAPDH	Invitrogen	AM4300	1:100000	Mouse
Goat anti-Rabbit IgG (H+L) Secondary Ab, HRP	Pierce	31460	1:6000	Goat
Goat anti-Mouse IgG (H+L) Secondary Ab, HRP	Pierce	31430	1:6000	Goat

Supplementary Table 2. Amplification primers used in RT-qPCR assays.

Gene name	Primer Forward (5’-3’)	Primer Reverse (5’-3’)
HLA-A	AGATACACCTGCCATGTGCAGC	GATCACAGCTCCAAGGAGAACC
HLA-B	CTGCTGTGATGTGTAGGAGGAAG	GCTGTGAGAGACACATCAGAGC
HLA-C	GGAGACACAGAAGTACAAGCGC	ACATCCTCTGGAGGGTGTGAGA
B2M	CCACTGAAAAAGATGAGTATGCCT	CCAATCCAAATGCGGCATCTTCA
TAP1	GCAGTCAACTCCTGGACCACTA	CAAGGTTCCCACTGCTTACAGC
TAP2	ATGCCCTTCACAATAGCAGCGG	CCAAAACTGCGAACGGTCTGCA
KLF2	CCAAGAGTTCGCATCTGAAGGC	CCGTGTGCTTTCGGTAGTGGC
CDKN1A	AGGTGGACCTGGAGACTCTCAG	TCCTCTTGGAGAAGATCAGCCG
TBP	GAATATAATCCCAAGCGGTTTG	ACTTCACATCACAGCTCCCC

Supplementary Table 3. Cell lines used in this study and their major driver genetic alterations.

Cell line	Tumor type	Major driver alterations
IMR-32	Neuroblastoma	*MYCN* amplification; 1p loss; 17q gain
Ishikawa	Endometrioid cancer	*PTEN* loss; *TP53* mutation, *PIK3R1* mutation
LNCaP	Prostate cancer	*AR* T877A; *PTEN* loss; *PIK3CA* mutation
ARK1	Serous endometrial cancer	*PIK3CA* mutation, *HER2* overexpression
A549	Non-small cell lung cancer	*KRAS* G12S; *STK11* loss; *KEAP1* mutation
SW620	Colorectal cancer	*KRAS* G12V; *APC* truncation; *TP53* mutation; *SMAD4* mutation
