# Supplementary material for: MAPK ERK5 is a novel regulator of MHC-I in cancer cells
Source: Cell Commun Signal. 2026 Mar 9;24:227. doi: 10.1186/s12964-026-02780-9 (PMC13085394; doi:10.1186/s12964-026-02780-9)
Supplement: Supplementary file 3 — Supplementary Material 3. [file 12964_2026_2780_MOESM3_ESM.pdf]

Figure 1D

Neuroblastoma IMR-32 cells

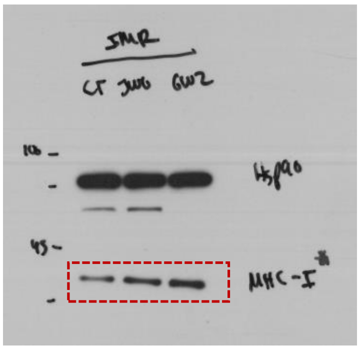

MHC-I

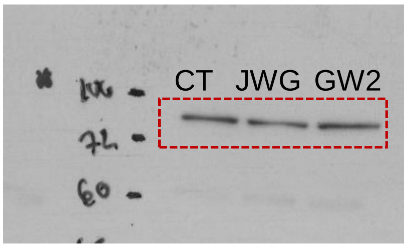

Hsp90

Endometrial Ishikawa cells

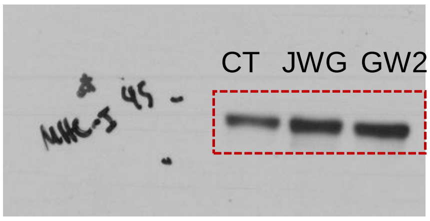

MHC-I

CT JWG GW2

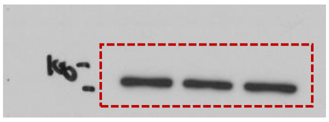

Hsp90

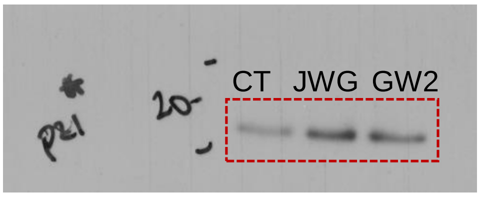

p21

Prostate cancer LNCaP cells

CT JWG GW2

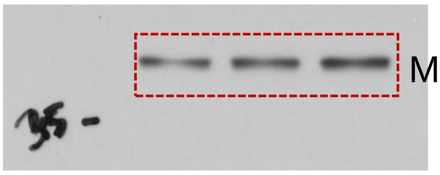

MHC-I

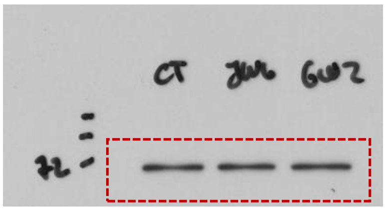

Hsp90

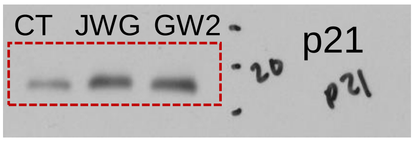

Endometrial ARK1 cells

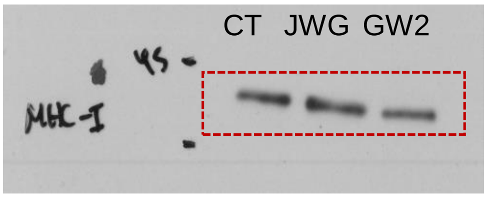

MHC-I

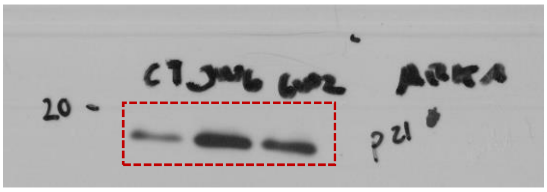

p21

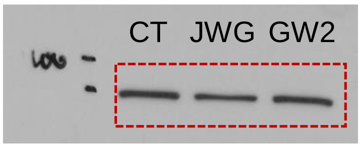

Hsp90

Vinas-Casas et al. Uncropped blots.

Figure 1D

Non-small cell lung cancer A549 cells

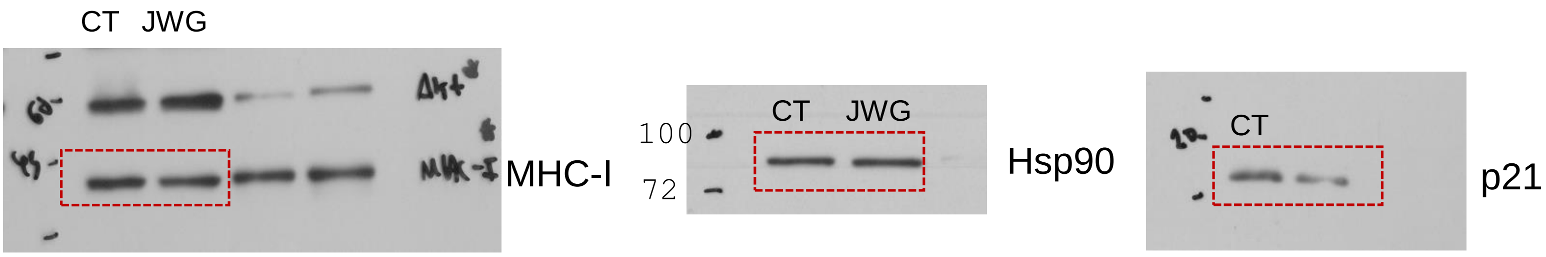

Colorectal cancer SW620 cells

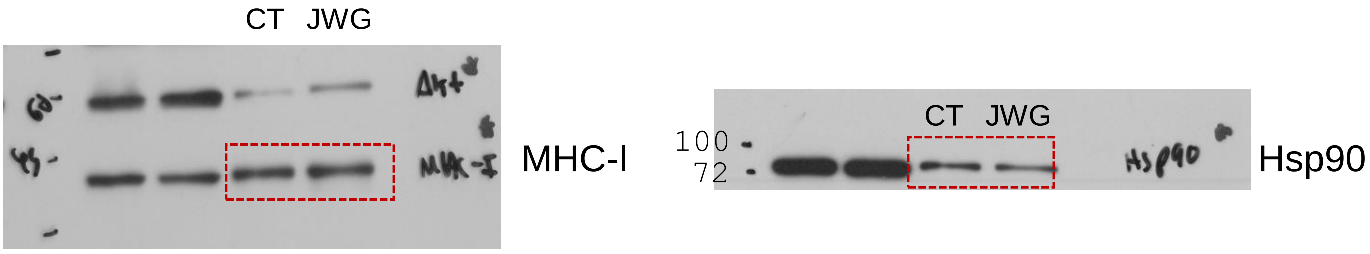

Figure 1F

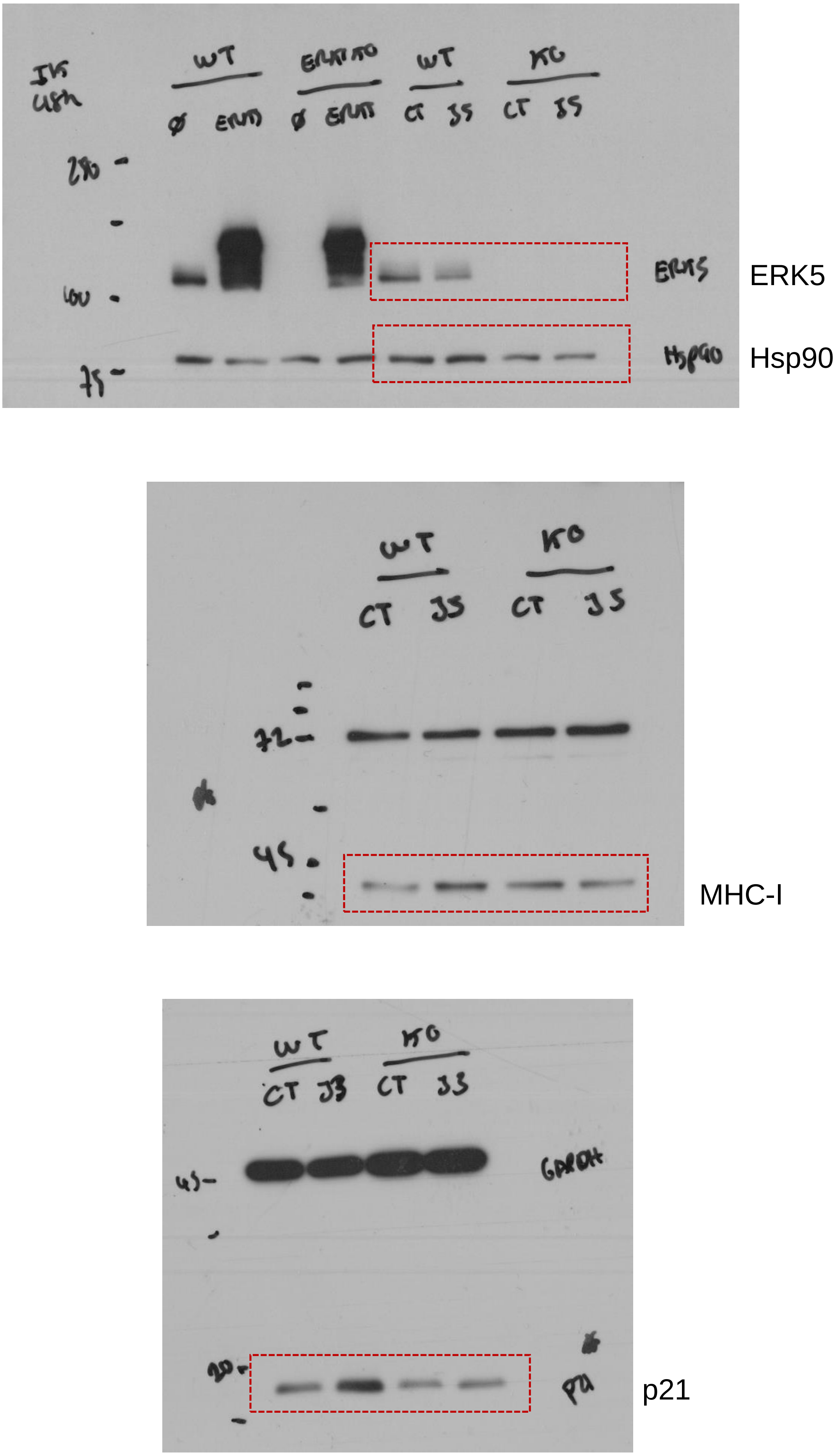

Vinas-Casas et al. Uncropped blots.

Figure 1F

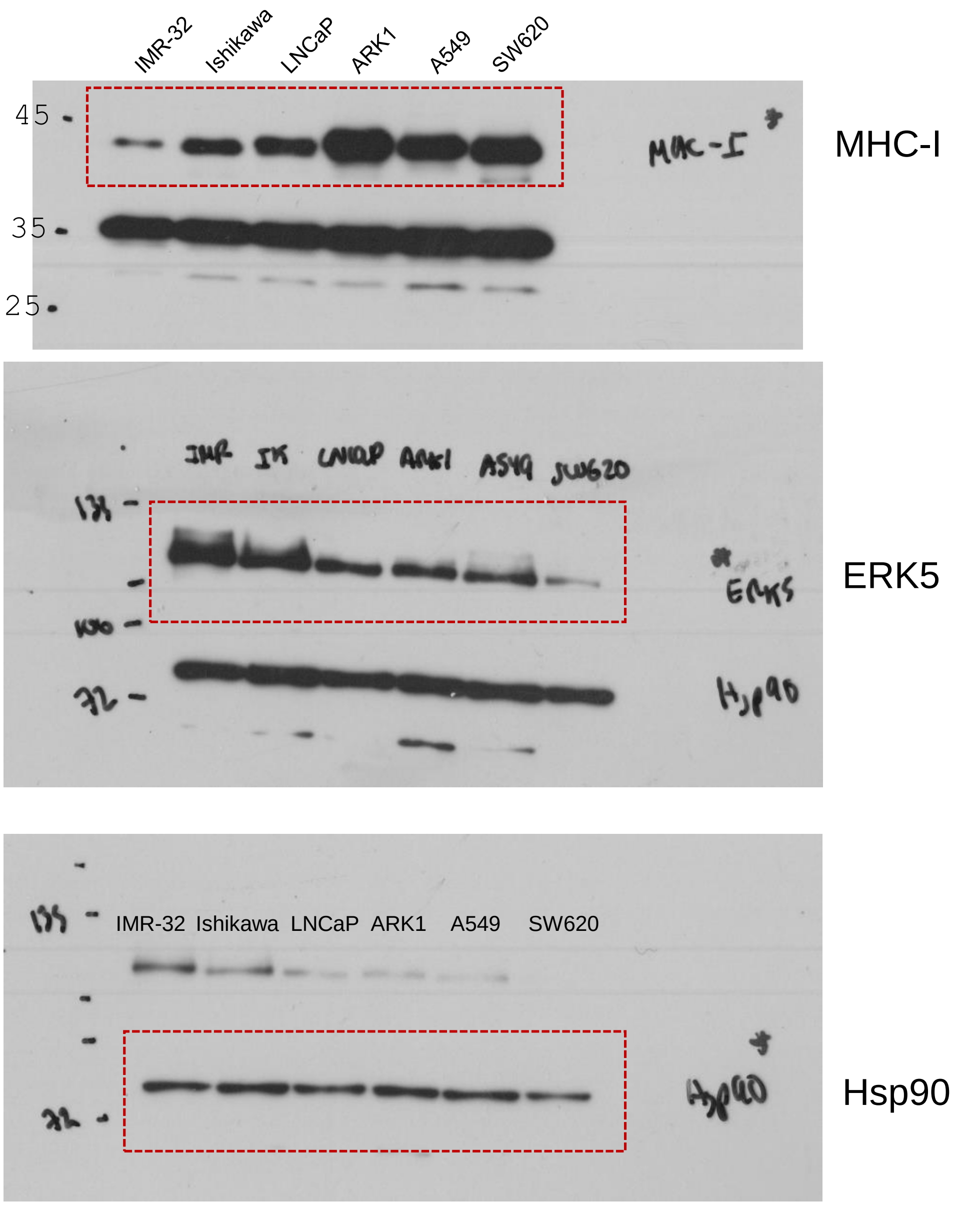

Vinas-Casas et al. Uncropped blots.

Figure 2A

Neuroblastoma IMR-32 cells

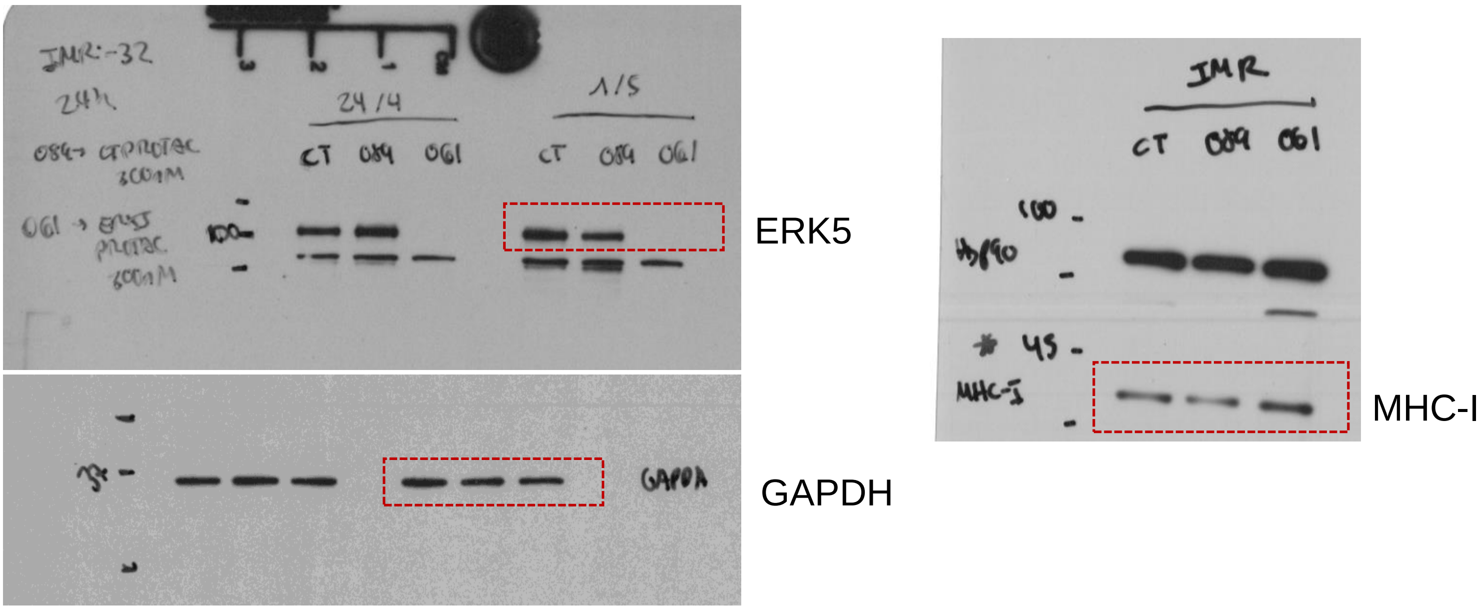

Figure 2B

Endometrial Ishikawa cells

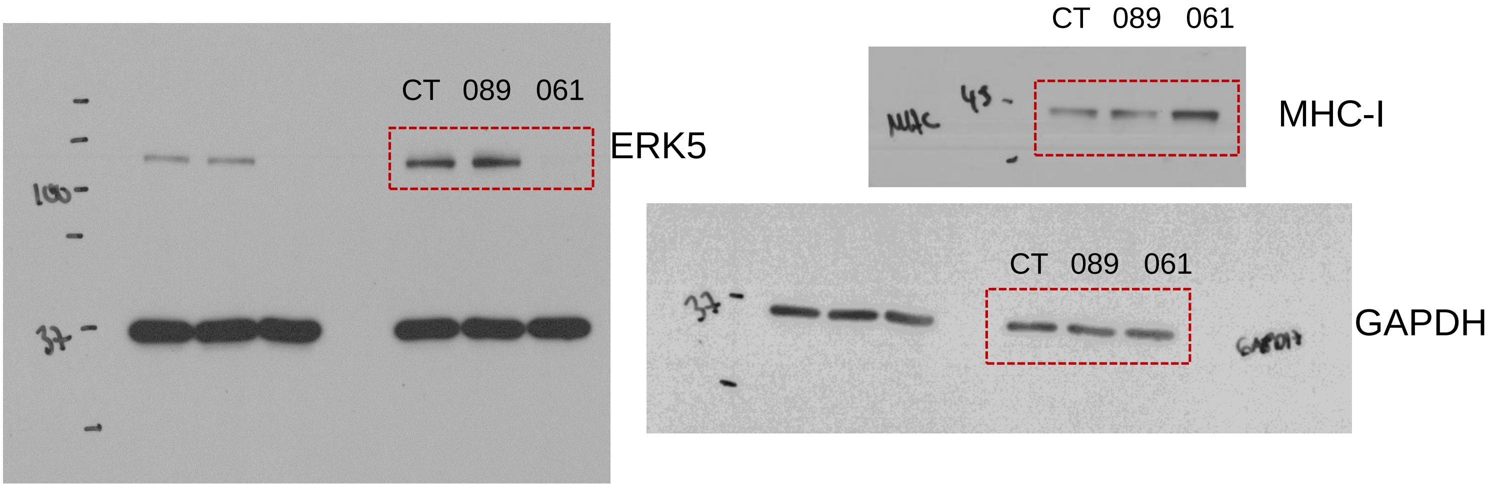

Figure 2C

Prostate cancer LNCaP cells

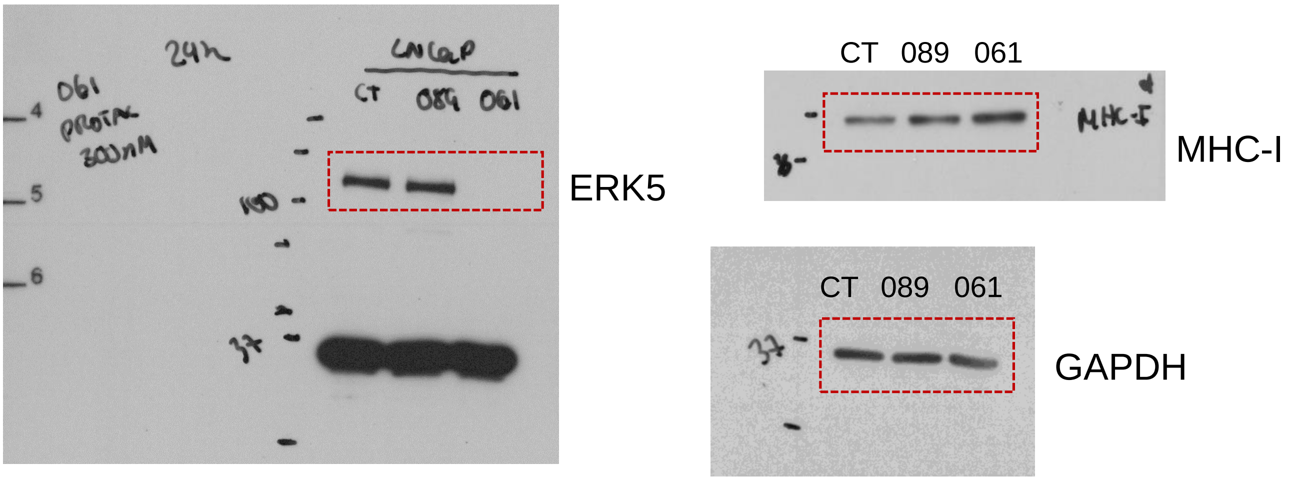

Vinas-Casas et al. Uncropped blots.

Figure 2D

Endometrial ARK1 cells

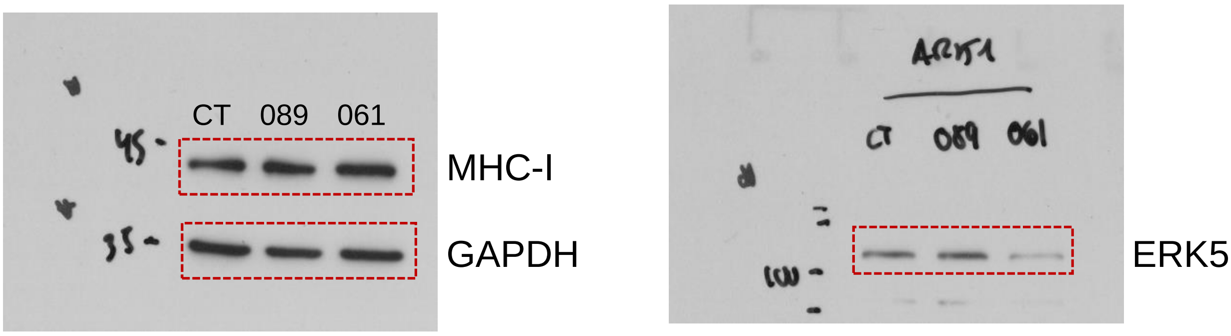

Non-small cell lung cancer A549 cells

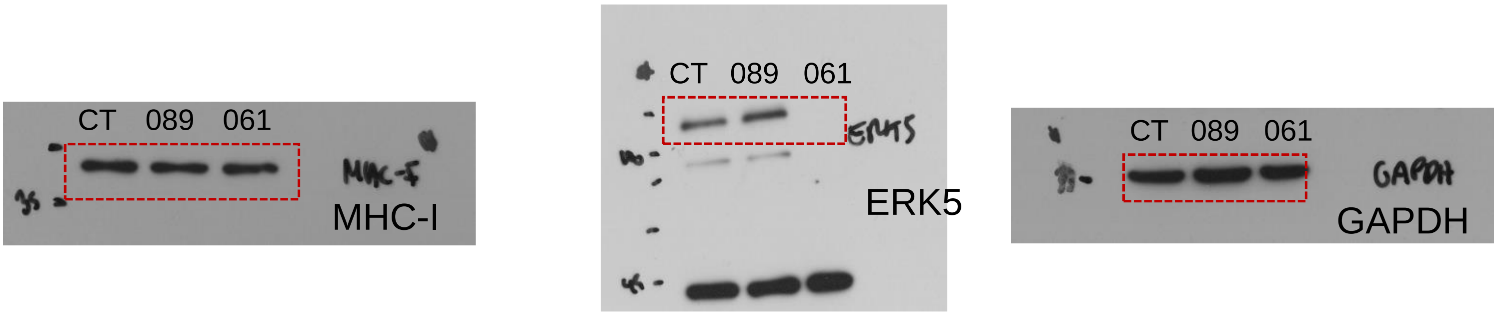

Colorectal cancer SW620 cells

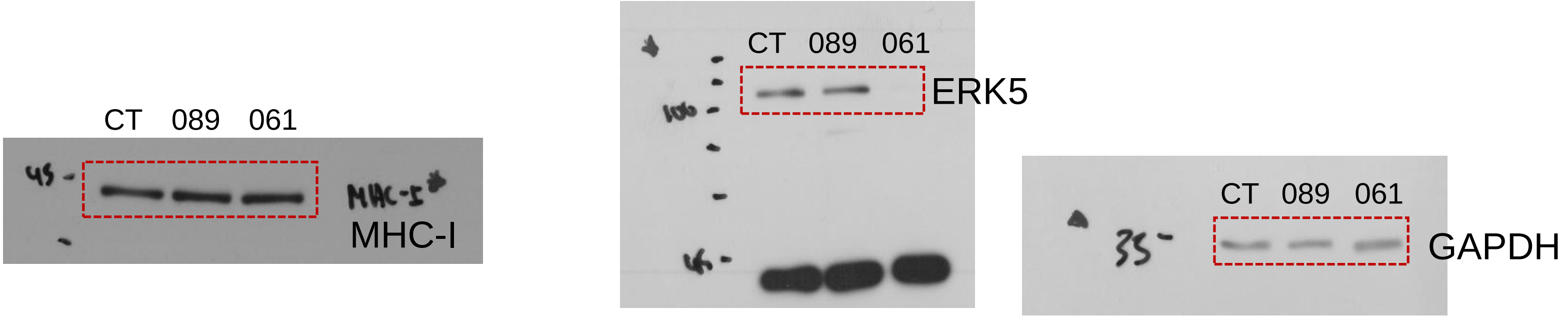

Vinas-Casas et al. Uncropped blots.

Figure 2E

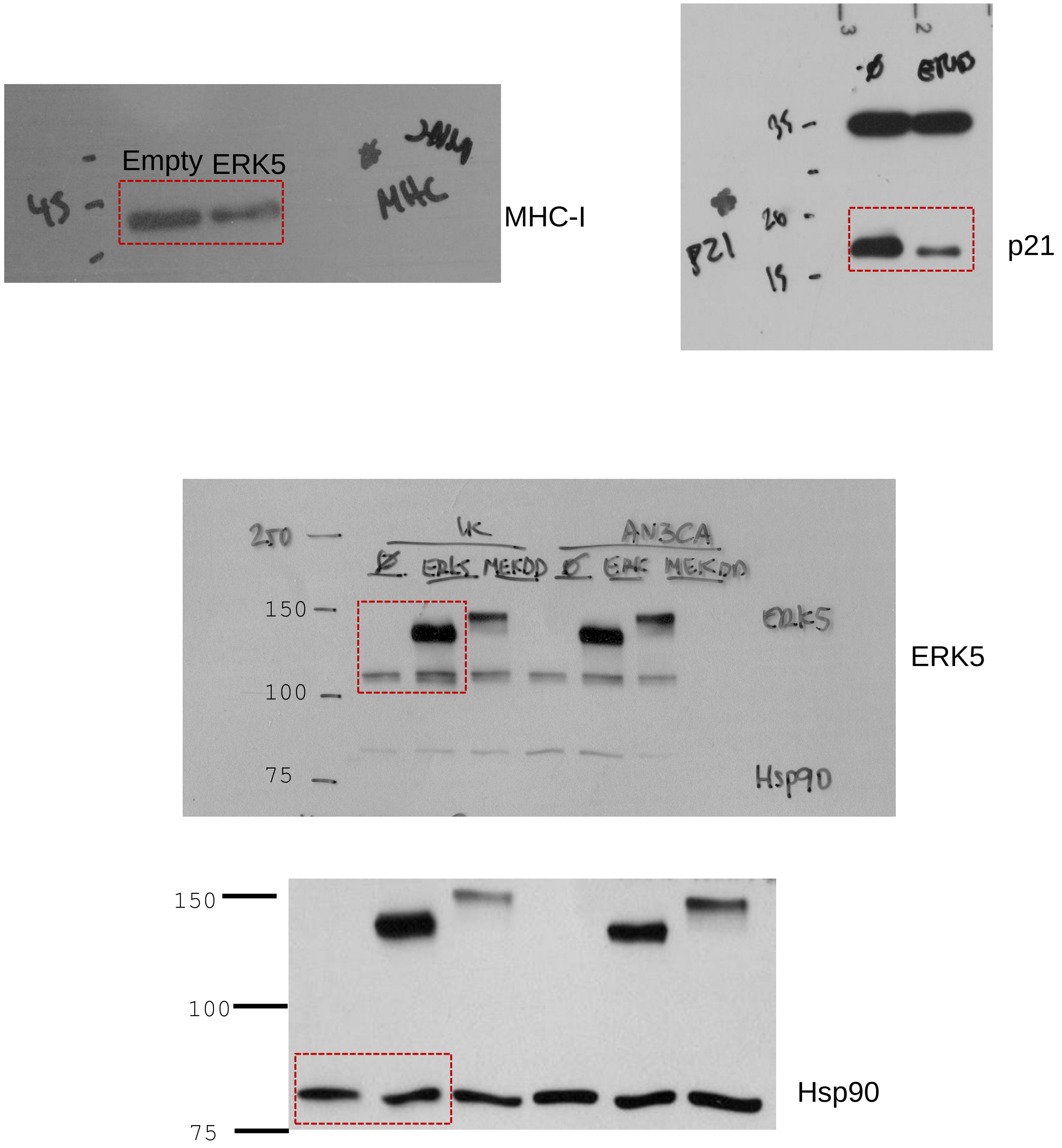

Figure 3B

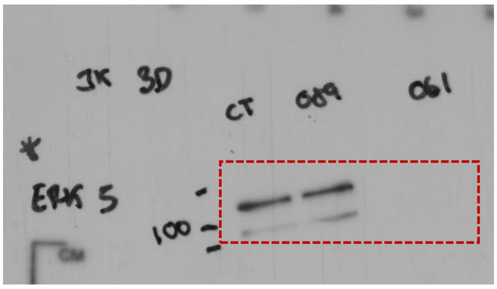

ERK5

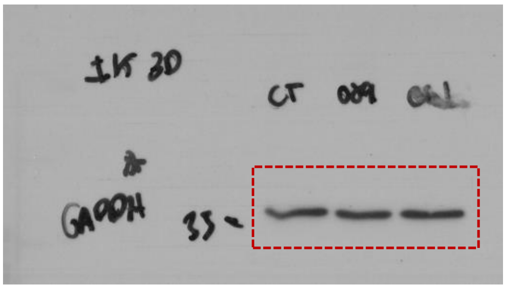

GAPDH

Vinas-Casas et al. Uncropped blots.

Figure 4F

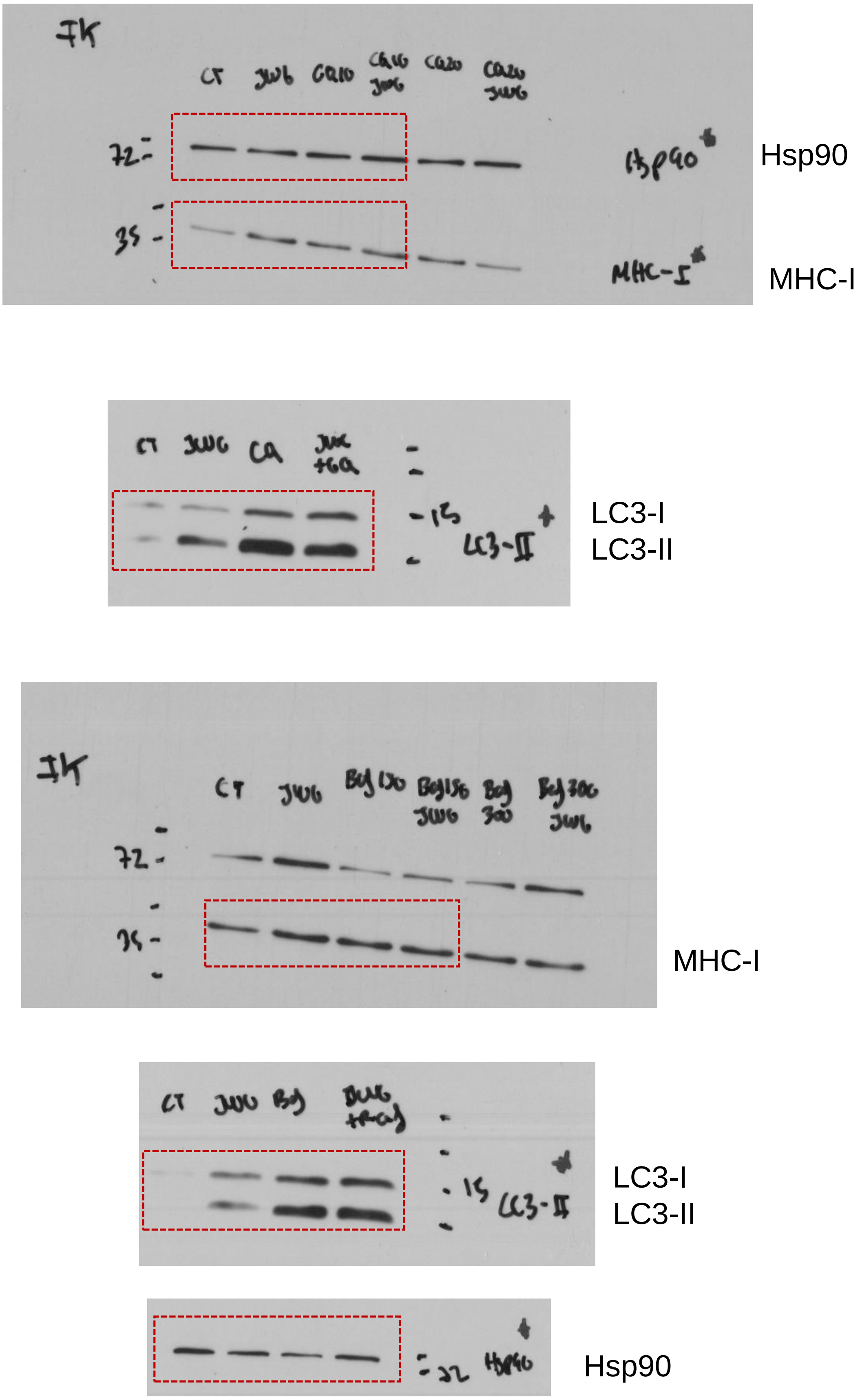

Vinas-Casas et al. Uncropped blots.

Supplementary Figure 1

Neuroblastoma IMR-32 cells

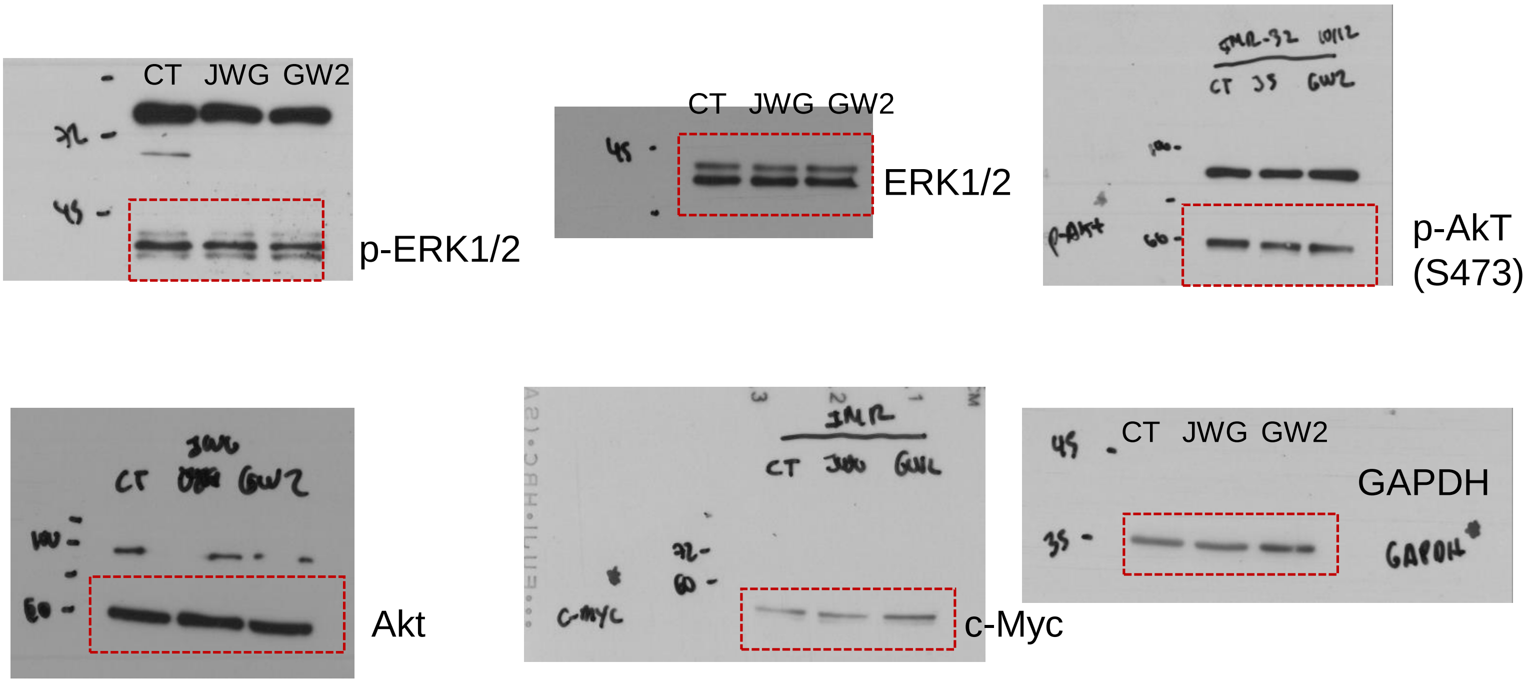

Endometrial Ishikawa cells

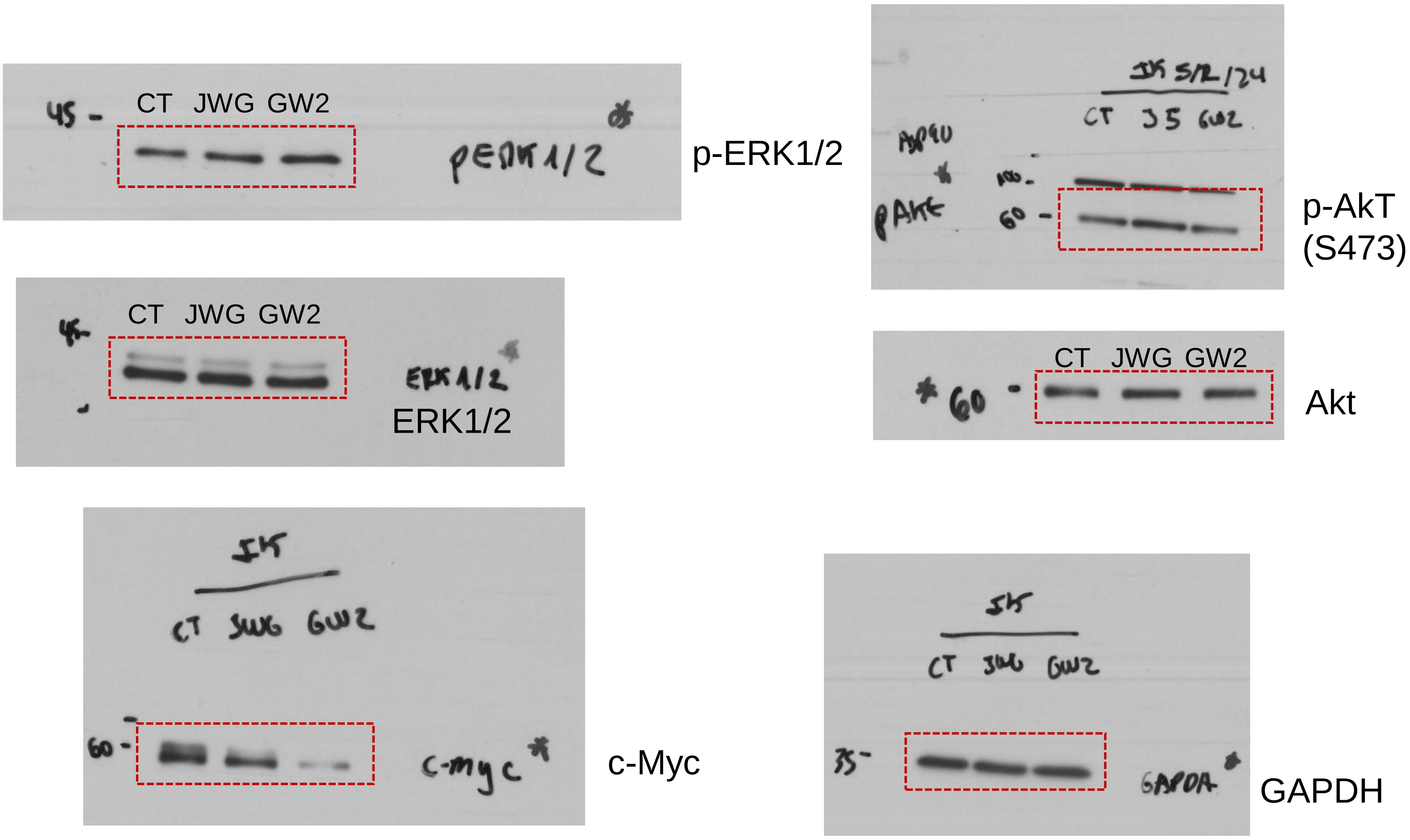

Vinas-Casas et al. Uncropped blots.

Supplementary Figure 1

Prostate cancer LNCaP cells

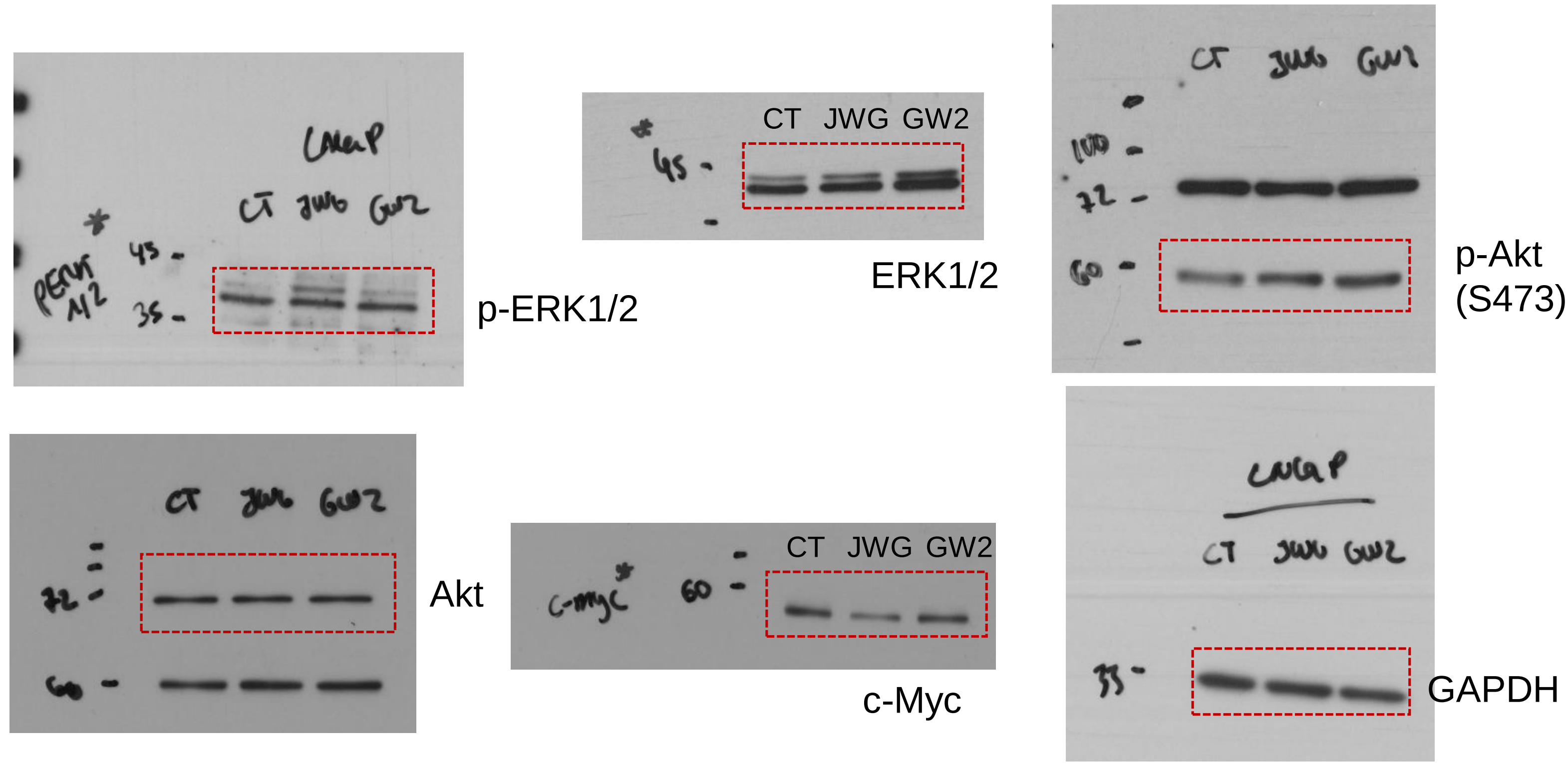

Endometrial ARK1 cells

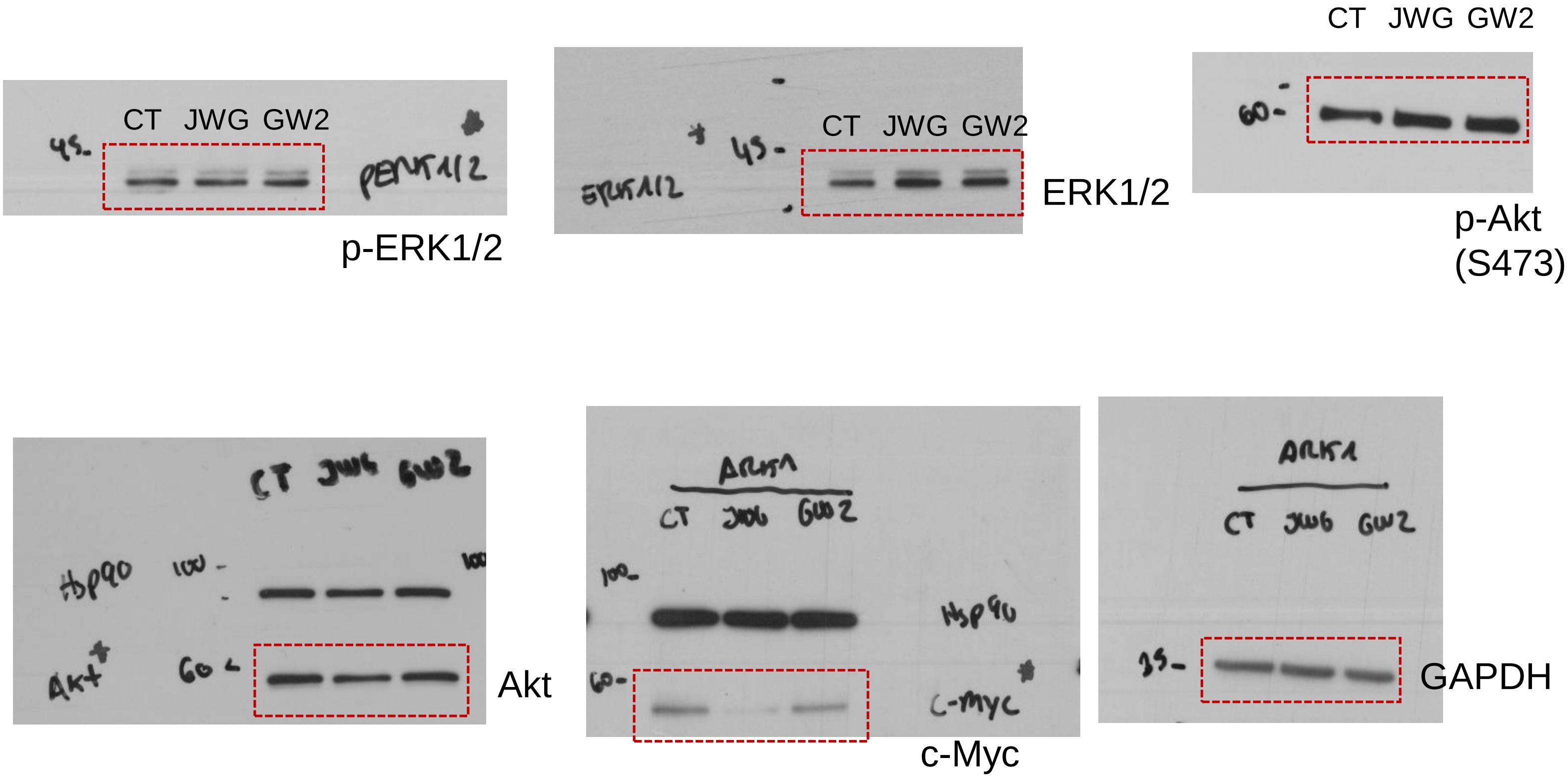

Vinas-Casas et al. Uncropped blots.

Supplementary Figure 1

Non-small cell lung cancer A549 cells

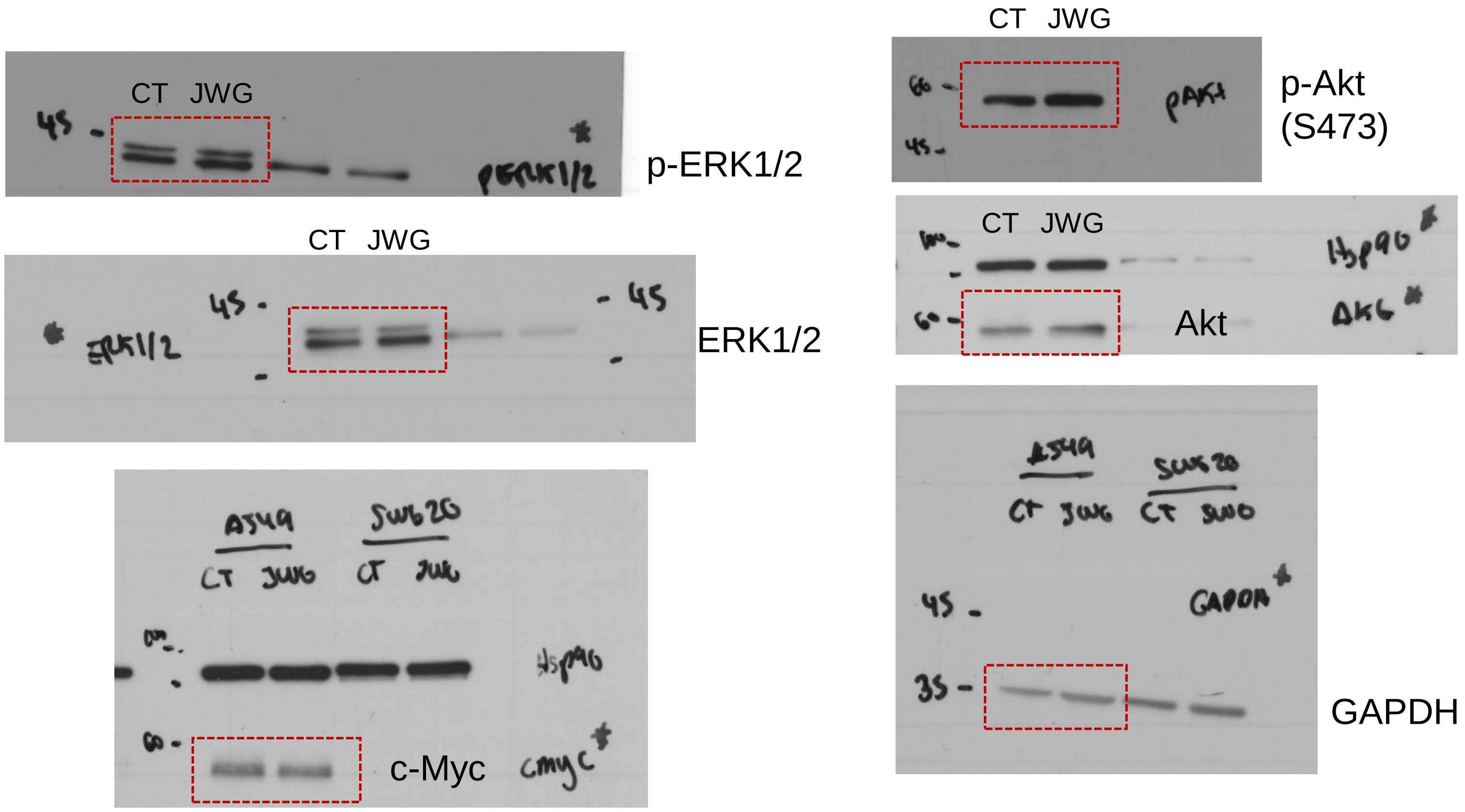

Colorectal cancer SW620 cells

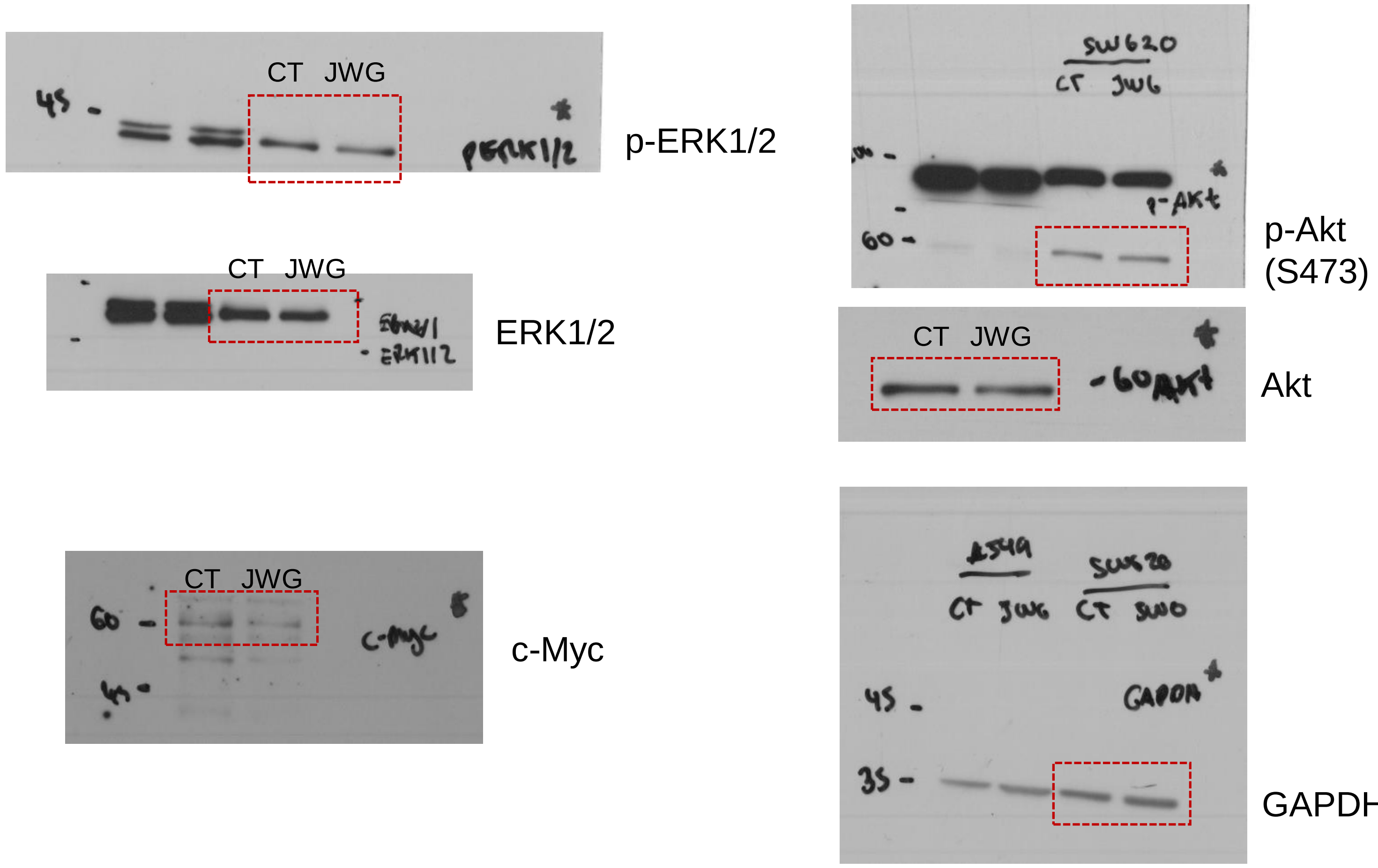

Vinas-Casas et al. Uncropped blots.

Supplementary Figure 2

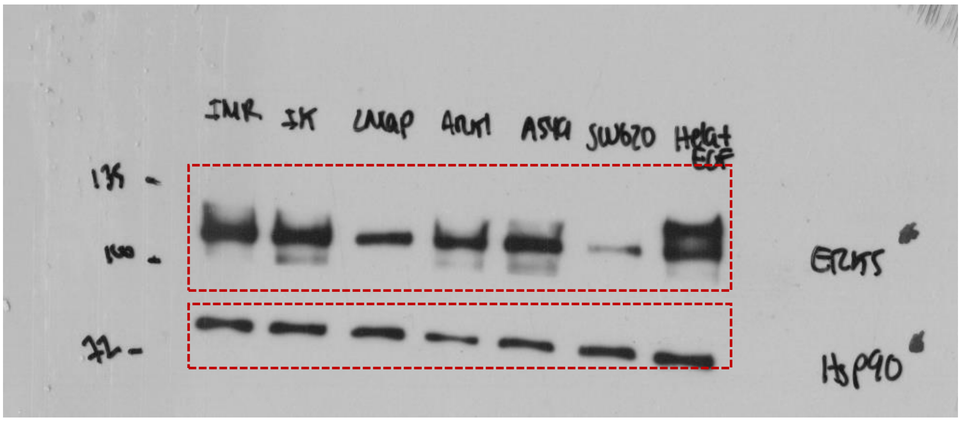

ERK5

Hsp90

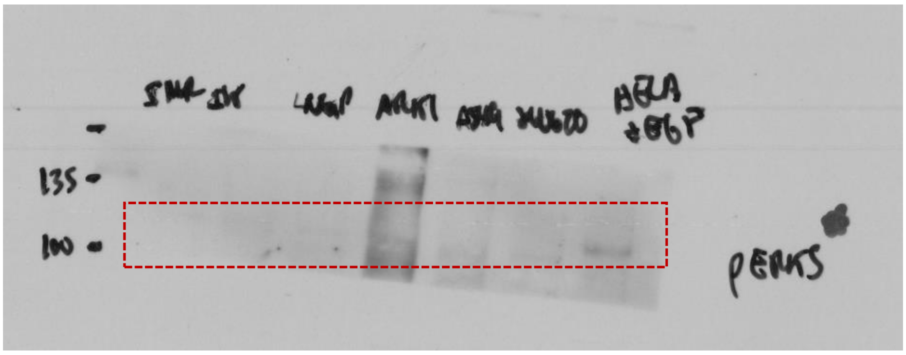

p-ERK5

Supplementary Figure 3

Neuroblastoma IMR-32 cells

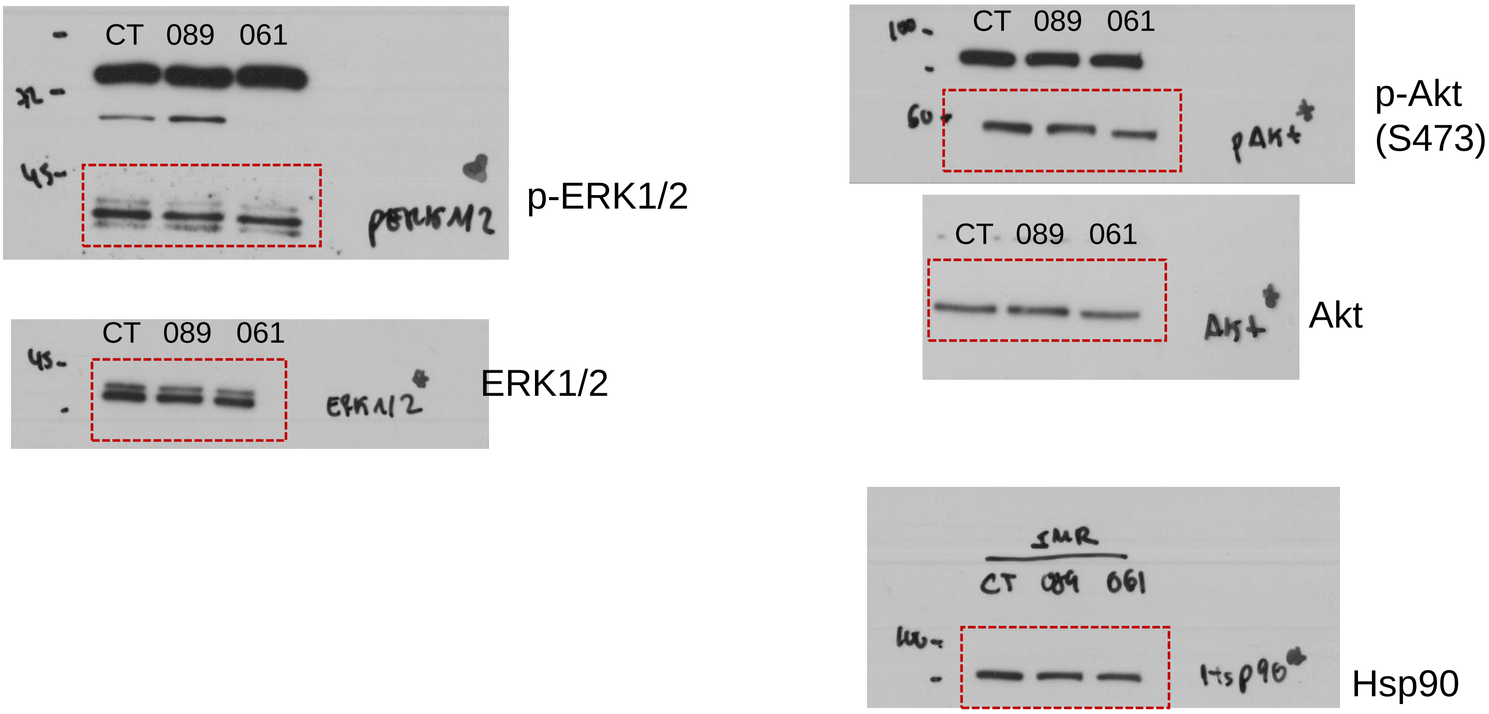

Endometrial Ishikawa cells

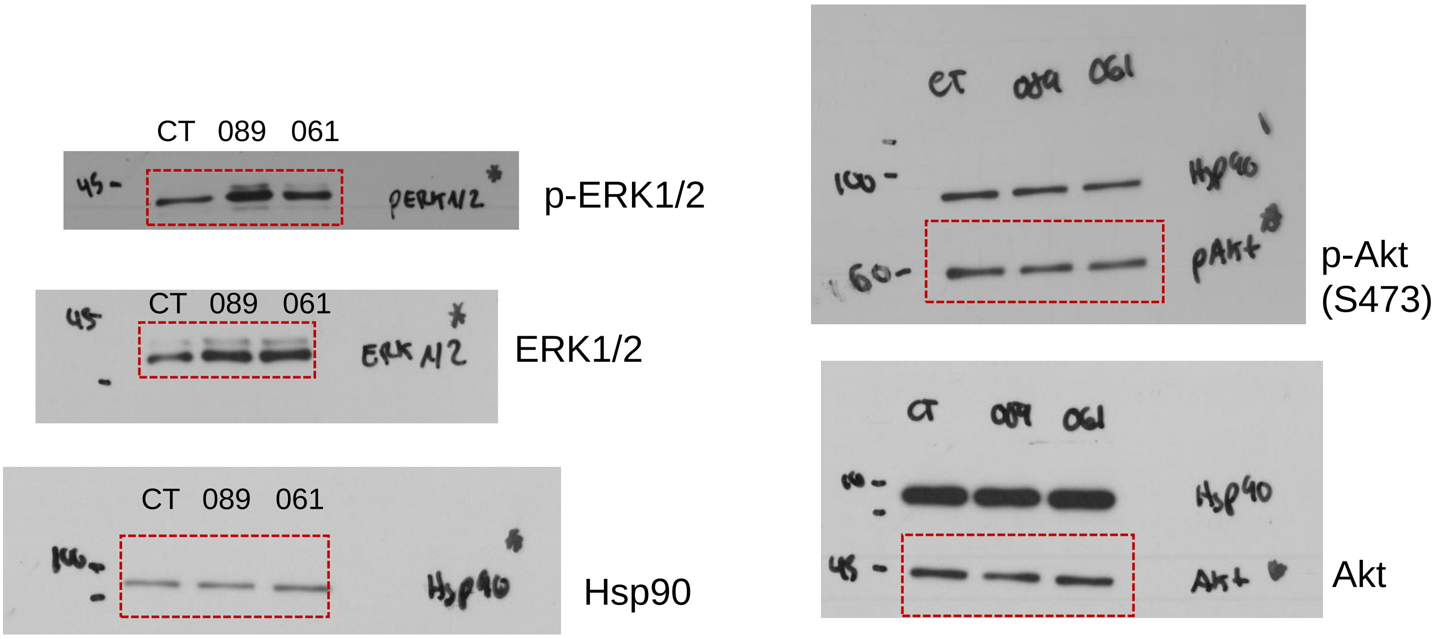

Supplementary Figure 3

Prostate cancer LNCaP cells

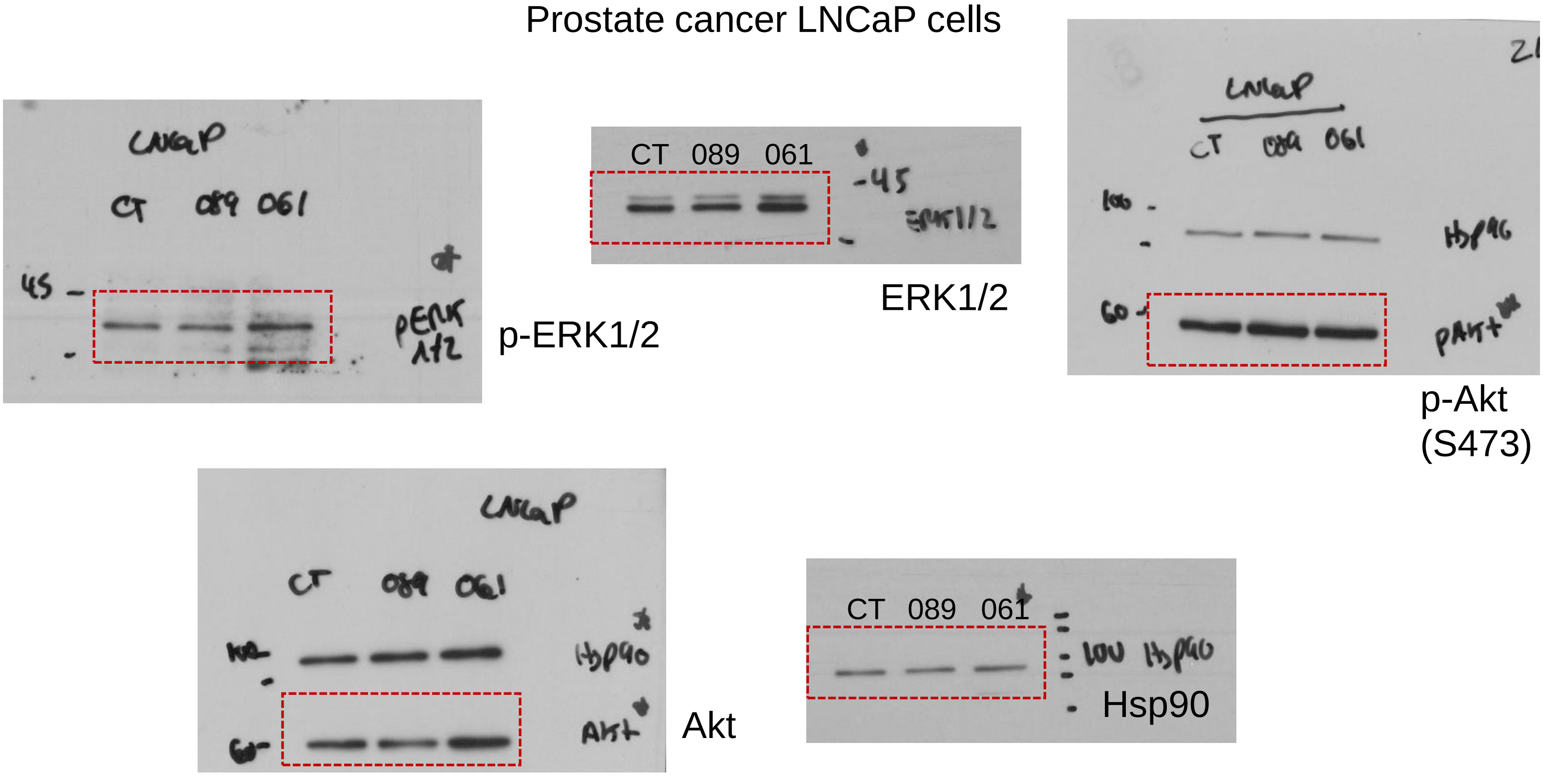

Endometrial ARK1 cells

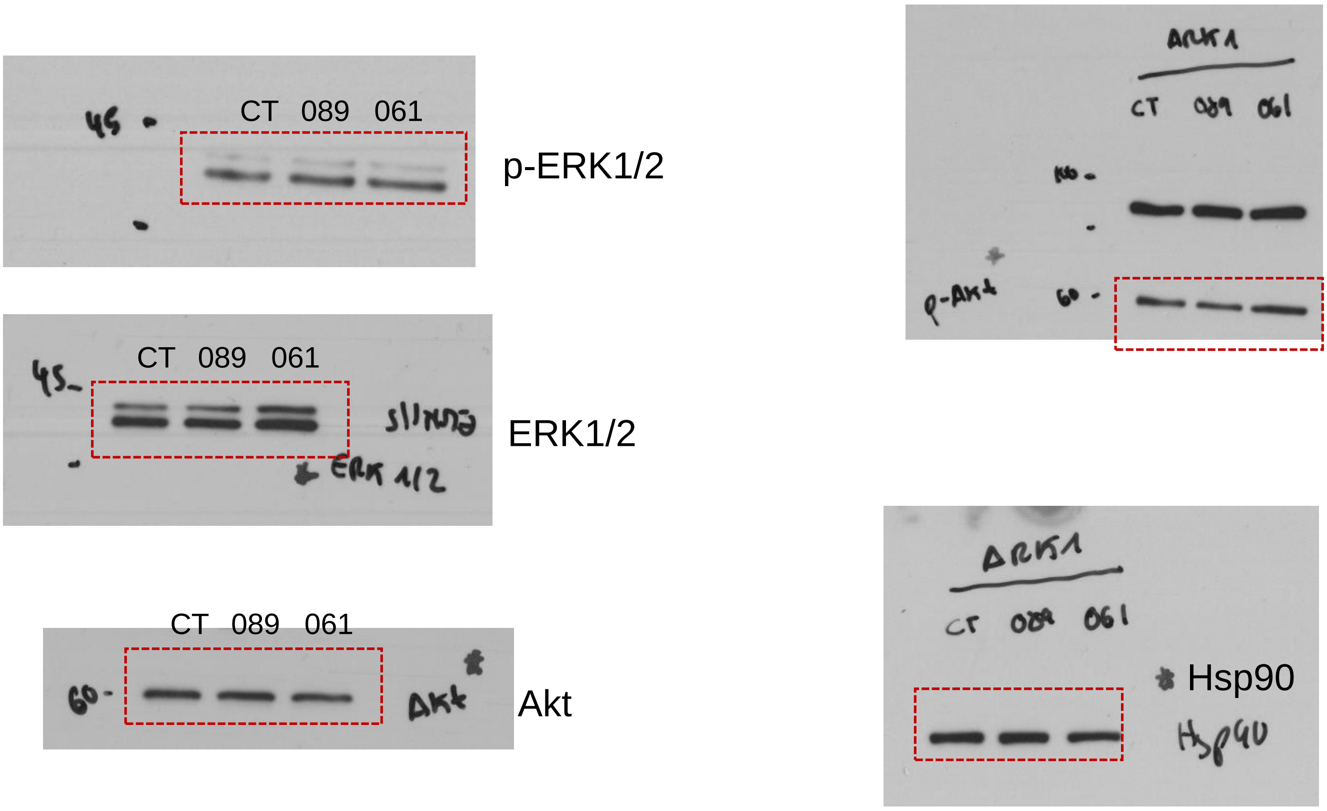

Vinas-Casas et al. Uncropped blots.

Supplementary Figure 3

Non-small cell lung cancer A549 cells

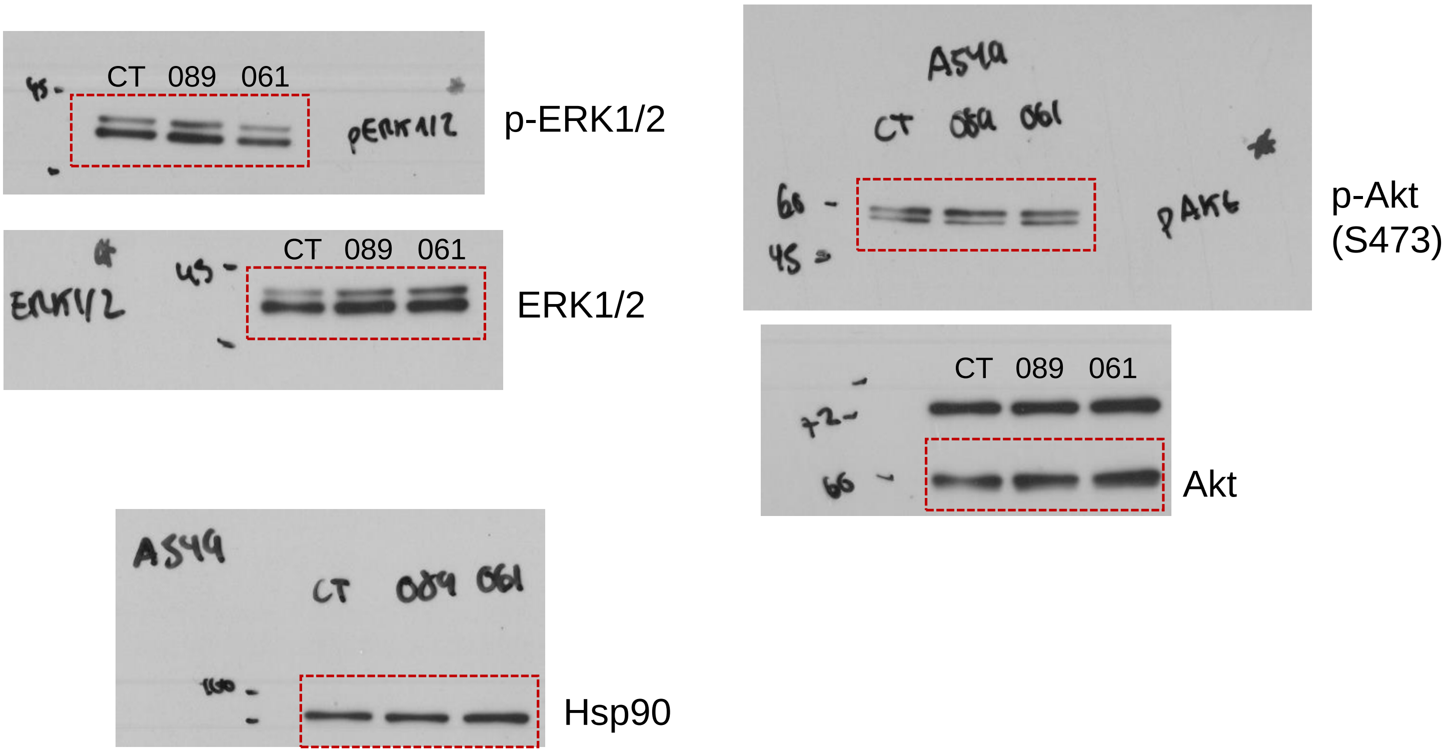

Colorectal cancer SW620 cells

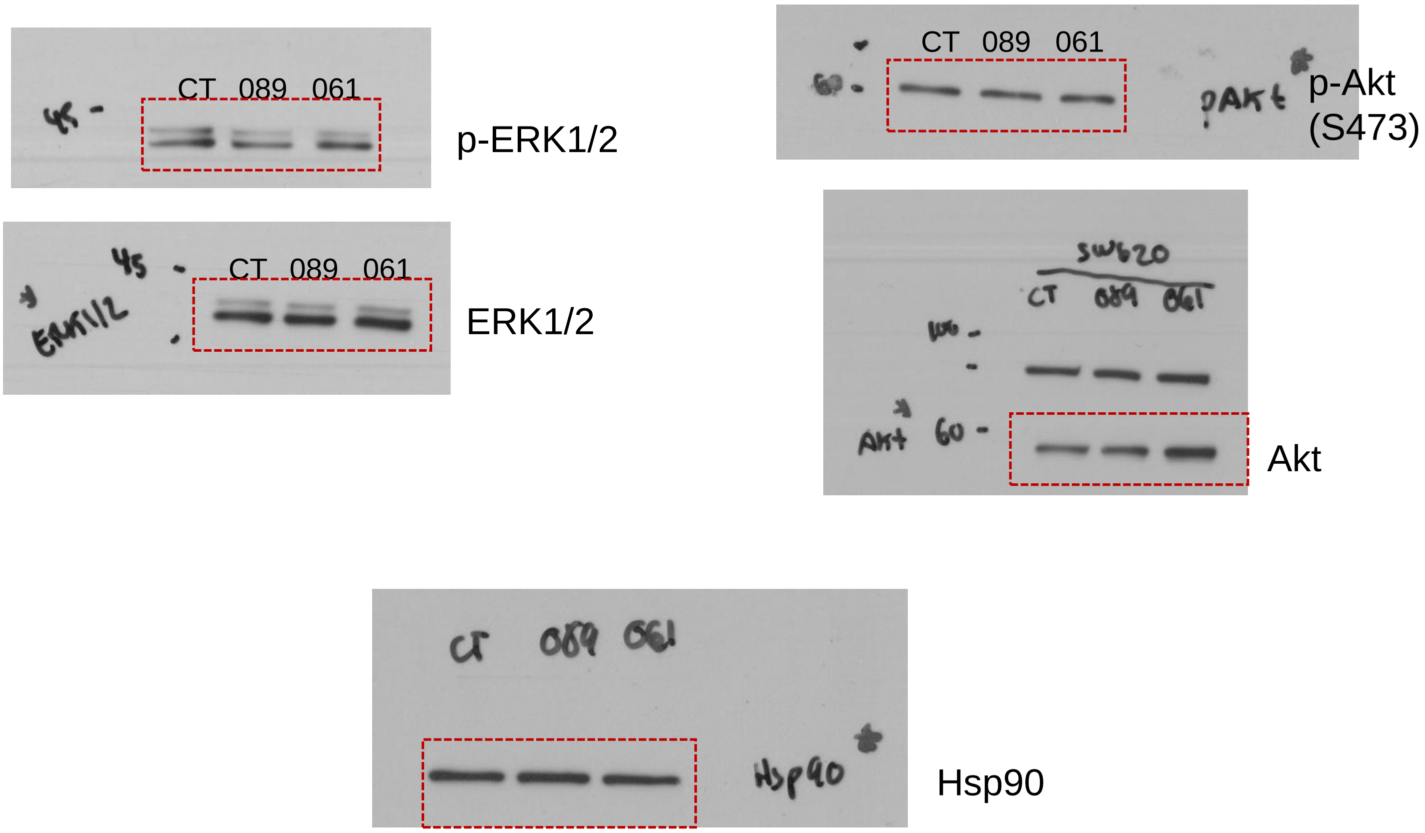

Vinas-Casas et al. Uncropped blots.
